# The Prevalence and Characterization of Fecal Extended-Spectrum-Beta-Lactamase-Producing *Escherichia coli* Isolated from Pigs on Farms of Different Sizes in Latvia

**DOI:** 10.3390/antibiotics10091099

**Published:** 2021-09-11

**Authors:** Daiga Gāliņa, Andris Balins, Anda Valdovska

**Affiliations:** 1 Faculty of Veterinary Medicine, Latvia University of Life Sciences and Technologies, K. Helmana iela 8, LV-3004 Jelgava, Latvia; anda.valdovska@llu.lv; 2 Scientific Laboratory of Biotechnology, Latvia University of Life Sciences and Technologies, Liela iela 2, LV-3001 Jelgava, Latvia; andris.balins@llu.lv

**Keywords:** ESBL, pig farming, multidrug-resistant, CTX-M, SHV, TEM

## Abstract

The aim of this study was to determine the prevalence of fecal ESBL-producing *Escherichia coli* (*E. coli*) in pigs on large and small farms in Latvia, to characterize beta-lactamase genes and establish an antimicrobial resistance profile. Fecal samples (n = 615) were collected from 4-week, 5-week, 6-week, 8-week, 12-week and 20-week-old piglets, pigs and sows on four large farms (L1, L2, L3, L4) and three small farms (S1, S2, S3) in Latvia. ChromArt ESBL agar and combination disc tests were used for the screening and confirmation of ESBL-producing *E. coli*. The antimicrobial resistance was determined by the disc diffusion method and ESBL genes were determined by polymerase chain reaction (PCR). Subsequently, ESBL-producing *E*. *coli* was confirmed on three large farms, L1 (64.3%), L2 (29.9%), L3 (10.7%) and one small farm, S1 (47.5%); n = 144 (23.4%). The prevalence of ESBL-producing *E. coli* differed considerably between the large and small farm groups (26.9% vs. 12.7%). Of ESBL *E. coli* isolates, 96% were multidrug-resistant (MDR), demonstrating there were more extensive MDR phenotypes on large farms. The distribution of ESBL genes was *bla*_TEM_ (94%), *bla*_CTX-M_ (86%) and *bla*_SHV_ (48%). On the small farm, *bla*_SHV_ dominated, thus demonstrating a positive association with resistance to amoxicillin-clavulanic acid, ceftazidime and cefixime, while on the large farms, *bla*_CTX-M_ with a positive association to cephalexin and several non-beta lactam antibiotics dominated. The results indicated the prevalence of a broad variety of ESBL-producing *E. coli* among the small and large farms, putting the larger farms at a higher risk. Individual monitoring of ESBL and their antimicrobial resistance could be an important step in revealing hazardous MDR ESBL-producing *E. coli* strains and reviewing the management of antibiotic use.

## 1. Introduction

Antimicrobial resistance is one of the major global public health concerns currently facing humanity. The alarming situation has been exacerbated by the fact that the “pipeline of new antibiotics is dry”. In Europe, an estimated 70% of antibiotics are used in the animal sector, thus increasing the interest in antibiotic consumption in this sector [1]. According to global trends in antibiotic use for food animals, the highest consumption of antimicrobials per kilogram of an animal is in pigs, compared to chickens and cattle [2]. In high-income countries, intensive pig production dominates and pigs are generally kept in indoor systems in which there is limited space, few opportunities to express natural behaviors, and many stressors such as weaning, moving, mixing, surgical procedures, and environmental conditions that increase the risk of production diseases and infections. Digestive problems in early-weaned piglets is the main reason why piglets receive antibiotics around weaning and post-weaning periods [3]. Most of the antibiotics used in pigs are the same or belong to the same classes as those used in human medicine [4]. According to a report from the European Medicines Agency, tetracycline and beta-lactam antibiotics are the most sold antibiotics for food-producing animals in European countries [5,6]. Moreover, extended-spectrum penicillin accounts for the major proportion of penicillin subclasses [5]. The intensive use and misuse of beta-lactam antibiotics, which are critically important in human medicine, has resulted in the spread of extended spectrum beta-lactamase (ESBL)-producing bacteria in pigs. ESBLs are able to confer resistance to penicillin, first-, second-, and third-generation cephalosporin, and monobactam. Most ESBL genes are located in the mobile genetic elements; therefore, horizontal gene transfer has promoted the spread of antibiotic resistance genes among strains and species [7]. In addition, ESBL-producing *Escherichia coli* (*E. coli*) has often been found to be co-resistant to other antibiotic classes [8]. According to WHO, ESBL-producing bacteria are one of the most serious and critical global threats of the 21st century [9].

On the one hand, programs for routine monitoring of antimicrobial resistance have disclosed a low prevalence of ESBL producers among indicator *E. coli* from pigs in Europe, including Latvia; on the other hand, specific monitoring programs have used selective media and disclosed a highly varying prevalence of presumptive ESBL producers—from very low to extremely high, depending on the reporting country [4,10,11]. In Latvia, the prevalence of ESBL-producing *E. coli* has been reported as high, which is also the European average level [4]. This alarming level for the prevalence of ESBL producers calls for further examination of the situation. Large farms with high numbers of pigs may have a higher probability of infection and production diseases, which can lead to an increase in antibiotic treatment [12]. Furthermore, the availability and administration of antimicrobials can differ depending on the farm size and this may have an impact on the development of resistance genes [13]. To limit the spread of ESBL-producing *E. coli*, it is important to determine the presence of ESBL in large and small farms, and to consider the prevalence level in pigs at different ages. There is a lack of information on differences in resistant genes and phenotypic resistance in large and small farms in Latvia. Therefore, the aim of this study was to determine the prevalence of fecal ESBL-producing *E. coli* in pigs on large and small farms; to determine the distribution of the CTX-M, TEM, and SHV genotype in ESBL-producing *E. coli* isolates, and to characterize the antimicrobial resistance profile in pigs from Latvia.

## 2. Results

### 2.1. Prevalence of ESBL-Producing E. coli

Out of seven pig farms, the presence of fecal ESBL-producing *E. coli* was confirmed on four farms (L1, L2, L3 and S1; n = 144 (23.4%)). Among the large farms, the highest prevalence of ESBL-producing *E. coli* was observed on Farm L1 (64.3%), while among the small farms the prevalence on Farm S1 was 47.5%. The prevalence of ESBL-producing *E. coli* differed significantly between the large and small farm groups (26.9% vs. 12.7%, *p* = 0.029). Moreover, a notable difference (*p* < 0.05) was found between the large and small farms (Table 1).

Comparing the prevalence of fecal ESBL-producing *E. coli* in pigs of different ages, the presence of ESBL-producing *E. coli* was significantly higher in 4-week and 6-week-old piglets (28.2% and 35.1%, respectively) and in sows (41.6%), while the lowest prevalence of ESBL-producing *E. coli* was observed in pigs at 20 weeks (8.2%) (Table 1).

### 2.2. Resistance of ESBL-Producing E. coli Isolates Tested

**Figure 1:**
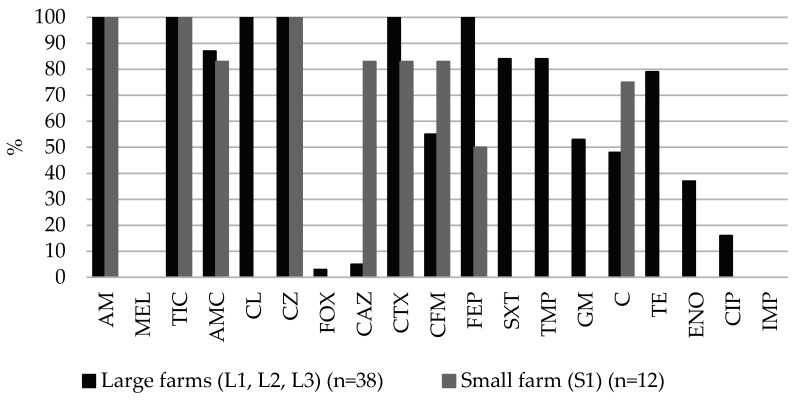
Prevalence of resistance in fecal ESBL-producing *E. coli* isolates from pigs on large and small farms. AM–ampicillin, MEL–mecillinam, TIC–ticarcillin, AMC–amoxicillin-clavulanic acid, CL–cefalexin, CZ–cefazolin, FOX–cefoxitin, CAZ–ceftazidime, CTX–cefotaxime, CFM–cefixime, FEP–cefepime, SXT–sulfamethoxazole-trimethoprim, TMP–trimethoprim, GM–gentamicin, C–chloramphenicol, TE–tetracycline, ENO–enrofloxacin, CIP–ciprofloxacin, IMP–imipenem.

On both the large and small farms, all of the ESBL-producing *E. coli* isolates (n = 50) were resistant to ampicillin, ticarcillin and cefazolin. On the large farms, the highest resistance of ESBL-producing *E. coli* was to cefalexin (100%), cefotaxime (100%), cefepime (100%), followed by amoxicillin-clavulanic acid (87%), trimethoprim (84%), sulfametaxazole-trimethoprim (84%), tetracycline (79%) and gentamicin (53%). On the small farm (S1), ESBL-producing *E. coli* was highly resistant to ceftazidime (83%), cefotaxime (83%), cefixime (83%), followed by chloramphenicol (75%) and cefepime (50%) (Figure 1). A statistically significant difference (*p* < 0.05) was found between the prevalence of resistance percentages of the large and small farms. On the large farms, there was higher (*p* < 0.05) resistance to cefalexin, cefepime, trimethoprim, sulfametaxazole-trimetoprim, gentamicin, tetracycline and enrofloxacin, while on the small farm (S1) there was higher resistance to ceftazidime. None of the tested ESBL-producing *E. coli* isolates were resistant to mecillinam and imipenem.

**Figure 2:**
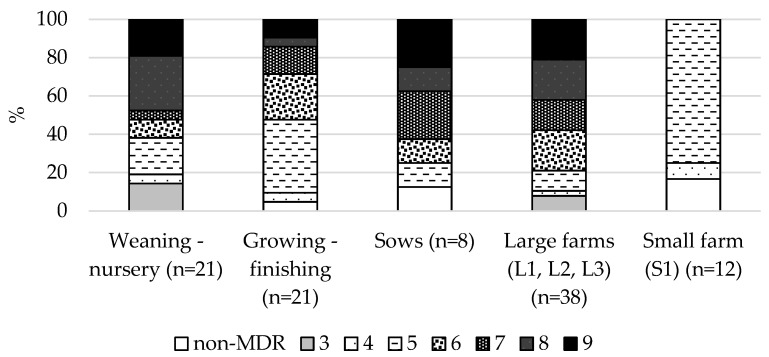
Proportion of different levels of MRD in 50 ESBL-producing *E. coli* isolates from pigs of different age stages and farms of different sizes.

Forty-eight of 50 (96%) fecal ESBL-producing *E. coli* isolates were classified as MDR according to Magiorakos et al., [14]. In a large proportion of the weaning-nursery piglets (47.6%) and sows (37.5%), ESBL-producing *E. coli* isolates were resistant to eight or more different antimicrobial categories, while there were less in the growing-finishing pigs (14.2%). ESBL-producing *E. coli* isolates from the large farms demonstrated more extensive MDR phenotypes; 79.0% of isolates were resistant to six or more antimicrobials. However, on the small farm (S1), 75.0% of ESBL-producing *E. coli* isolates were classified as MDR5, at the same time, none of them were resistant to six or more antimicrobials. Furthermore, 16.7% of ESBL-producing *E. coli* isolates were classified as non-MRD (Figure 2).

### 2.3. Distribution of Beta-Lactamase Gene(s) in ESBL-Producing E. coli

All the phenotypic confirmed fecal ESBL-producing *E. coli* isolates were confirmed genetically, and at least one ESBL gene was detected. The most frequently detected beta-lactamase gene in ESBL-producing *E. coli* isolates was *bla*_TEM_ (94%, n = 47), followed by *bla*_CTX-M_ (86%, n = 43) and *bla*_SHV_ (48%, n = 24). In the pig population, the most prevalent beta-lactamase genes were *bla*_TEM_ + *bla*_CTX-M_ in combination (42%, n = 21), followed by the combination of all three genes: *bla*_TEM_ + *bla*_SHV_ + *bla*_CTX-M_ (38%, n = 19).

**Figure 3:**
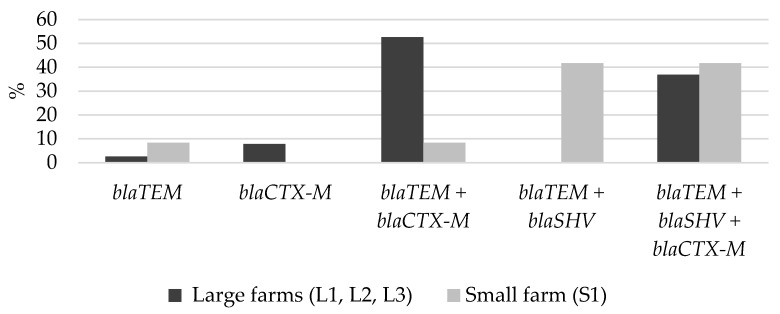
Distribution of beta-lactamase gene(s) on the large and small farms.

The distribution of beta-lactamase gene(s) on the large and small farms was different. On the small farm (S1), the *bla*_SHV_ combination with other beta-lactamase genes was detected considerably more frequently compared to the large farms (83.3% vs. 36.8%, *p* = 0.007), while *bla*_CTX-M_ alone, or in combination with other beta-lactamase genes, was more frequently detected on the large farms compared to the small one (97.4% vs. 50.0%, *p* < 0.001). On the large farms, the most prevalent beta-lactamase genes were *bla*_TEM_ + *bla*_CTX-M_ (52.6%, n = 20), compared to *bla*_TEM_ + *bla*_SHV_ (41.7%, n= 5) on the small farm (S1) (Figure 3).

### 2.4. Associations of Beta-Lactamase Gene(s) to Phenotypic Antimicrobial

The phenotype of AM-TIC-AMC-CL-CZ-CTX-CFM-FEP was the most commonly observed pattern of resistance to beta-lactams on the large farms (Table 2). This pattern most often contained all three beta-lactamase genes *bla*_TEM_ + *bla*_SHV_ + *bla*_CTX-M_ (52.9%, n = 9) or the combination of *bla*_TEM_ + *bla*_CTX-M_ (35, 3%, n = 6). The next most common phenotype that was observed was similar to the one above, but without resistance to cefixime. The phenotypes of AM-TIC-AMC-CZ-CAZ-CTX-CFM-FEP and AM-TIC-AMC-CZ-CAZ-CTX-CFM were the most commonly observed patterns on the small farm (S1). Interestingly, the phenotype AM-TIC-CZ was observed in two cases, one of these contained the *bla*_TEM_ gene, while the other contained *bla*_TEM_ + *bla*_SHV_ + *bla*_CTX-M_.

The presence of *bla*_SHV_ demonstrated a positive association with resistance to amoxicillin-clavulanic acid (OR = 6.79, 95% CI 1.39–66.10, *p* = 0.0083), ceftazidime (OR = 8.38, 95% CI 2.47–37.17; *p* = 0.0001) and cefixime (OR = 4.36, 95% CI 1.70–12.00; *p* = 0.0009). The presence of *bla*_CTX-M_ was positively associated with resistance to cefalexin (OR = 34.86, 95% CI 6.63–357.80; *p* = 0.0001). Furthermore, the presence of *bla*_CTX-M_ was positively associated (*p* < 0.05) with resistance to non-beta-lactam antibiotics—sulfamethoxazole-trimethoprim, trimethoprim, gentamicin, tetracycline and enrofloxacin (Table 3). No statistically significant association between the presence of *bla*_TEM_ and resistance to the antibiotics was observed.

## 3. Discussion

According to the program set up to monitor AMR in the EU Member states, the prevalence of the ESBL indicator *E. coli* was reported to be low (1.4%) in fecal samples from fattening pigs at slaughter. However, the specific monitoring program with supplementary testing discovered a high prevalence of presumptive ESBL-producing *E. coli* in the samples of fattening pigs (34.3%). It should be noted that the level of prevalence varies greatly (0.3–80.3%) among the EU countries [4]. In our study, the prevalence of ESBL-producing *E. coli* in pigs decreased during the period of growing and fattening. The highest amount of ESBL-producing *E. coli* was present in sows (41.6%), followed by weaned piglets (35.1%), and was much lower in fattening pigs (8.2%). It was previously reported that ESBL-producing *E. coli* carriage considerably decreases over the production cycle of pigs [15,16]. Interestingly, in the above study by the monitoring program of the EU, a much higher prevalence of ESBL-producing *E. coli* in fattening pigs was reported in Latvia, where the prevalence was 42.3% [4] in comparison with our results. One of the reasons could be the differences in the ESBL screening method. The method employed by us did not include a non-selective pre-enrichment step, which could affect the sensitivity of the method. A research study from Germany has shown that by including the enrichment procedure, ESBL-producing *E. coli* could be isolated twice as often [17]. In addition, a German study reported that during the transportation and the waiting period at the slaughterhouse, the probability of transmission of ESBL-producing *Enterobacteriacae* among pigs increased to nearly 30% [18]. In our study, samples were obtained on farms; thus, the testing method could result in a higher prevalence of ESBL-producing *E. coli*.

Our results demonstrated that farm size could be one of the risk factors for an increased occurrence of ESBL-producing *E. coli.* At the same time, this conclusion should be treated with caution, because farms with the highest prevalence represented both sizes: large (L1) and small (S1). It is more important to highlight the considerable differences observed on farms in general. Several studies on the risk factors of increased prevalence of ESBL-producing *E. coli* on pig farms have been carried out. The use of private water sources for pigs, lack of well-organized biosecurity (entrance hygiene, pest control, presence of pets, etc.) and hygiene (disinfection, detergent use), the high use of antibiotics in pig production, and in particular, the recent use of cephalosporins [19,20] are serious risk factors. Although our study did not include a detailed evaluation of risk factors for the spread of ESBL-producing *E. coli* due to the limited number of the farms available for our study, a causal relationship was observed between the increased occurrence of diarrhea and the wide use and misuse of beta-lactam antibiotics in pigs, resulting in an increased prevalence of ESBL. Based on the survey data, on farms L1 and S1, diarrhea in piglets of different ages has been a common problem for a long time; moreover, overuse of amoxicillin and enrofloxacin could be responsible for the selection of ESBL-producing bacteria. Amoxicillin is usually added to feed, but as it has low absorption and bioavailability [21], it creates high selective pressure on gut microbiota, which can promote antibiotic resistance [22]. A correlation between the use of beta-lactam antibiotics (amoxicillin) and the high prevalence rate of ESBL producers was also reported in another study [23]. In contrast, on the sampled two small farms (S2 and S3) and on one large farm (Farm L4), diarrhea was rarely observed. On Farms S2 and S3, antibiotics, and non-beta lactam antibiotics, were used rarely; a similar situation was observed on Farm L4, where antibiotics from the broad-spectrum penicillin group were only used sporadically. The role of fluoroquinolone in increasing the spread of ESBL has also been confirmed [24].

The phenotypic detection of antimicrobial resistance in ESBL-producing *E. coli* revealed significant differences in resistance between isolates from the large and small farms. With regard to beta-lactam antibiotics, the resistance to cephalexin, cefotaxime and cefepime was more commonly observed in pigs from the large farms, while resistance to ceftazidime was found in pigs from the small farm. Furthermore, resistance to the non-beta lactam antibiotics: trimethoprim, sulfametaxazole-trimetoprim, gentamicin, tetracycline, enrofloxacin was observed only in isolates obtained from the large farms. Phenotypic differences in resistance to beta-lactam antibiotics were discovered by the presence of different beta-lactamases coding genes. As known, ESBL-producing bacteria produce ESBLs enzymes that hydrolyze most third- and fourth-generation cephalosporins, thus explaining the observation of the high prevalence of resistance to cefotaxime (100%) and cefepime (100%). At the same time, a high prevalence of resistance to amoxicillin-clavulanic acid (87%) was observed in fecal ESBL-producing *E. coli* isolates. One of reasons for this phenomenon could be the production of OXA-1 beta lactamase [25]. Further investigations are needed to confirm this; in addition, this could also be due to the hyperproduction of target beta-lactamases or relative permeability [26]. Extended-spectrum SHV and TEM beta-lactamases show higher levels of hydrolytic activity for ceftazidime than cefotaxime [27], while a large number of CTX-M beta lactamases show only cefotaxime activity [28]. The co-existence of beta-lactamase genes, especially *bla*_CTX-M,_ with other genes, was explained by MDR phenotypes [29]. Resistance to mecillinam and imipenem was not observed in our study. Sporadic cases of ESBL-producing *E. coli* resistance to imipenem (carbapenems) obtained from pigs or pork in the EU have been reported in Germany [4] and Belgium [30]. Mecillinam belongs to the group of broad-spectrum penicillins and historically it has not been widely used in veterinary medicine, possibly due to its poor oral absorption; however, some authors believe that mecillinam has potential in veterinary medicine if administered parenterally [31]. No data are available on the ESBL-producing *E. coli* resistance to mecillinam in pigs, but several authors have reported the high susceptibility of ESBL-producing *E. coli* (96.9.2%) and MRD *E. coli* (96.5%) to mecillinam in humans [32]; therefore, since 2019, mecillinam has been included in category A, which is not authorized for use in veterinary medicine [33].

In contrast to ESBL-producing *E. coli* on the small farm, all of the ESBL-producing *E. coli* isolates from pigs on the large farms were classified as MDR; moreover, there were more extensive MDR phenotypes. This is in accordance with another study where these results were positively associated with the farm size, and a higher rate of MDR in *E. coli* on pig farms was reported [13]. No detailed information on the treatment management on the pig farms was available, but the higher usage of antibiotics in feed or water on the large farms in comparison to parenteral treatment on the small farm could be a credible explanation for higher and more extensive MDR isolates on the large farms. Moreover, the inclusion of zinc oxide as a feed additive could be an important factor in increasing extensive MDR phenotypes. High levels of zinc oxide supplementation may promote antimicrobial resistance and MDR in the pig’s gut [34,35]. In our study, the group of growing-finishing pigs showed a tendency to dominate over narrower phenotypes of MDR. Indeed, narrower phenotypes of MDR in growing-finishing pigs were associated with a decrease in external selection pressure due to considerably reduced or completely discontinued antibiotic use during the fattening period. Several studies have reported a significant reduction in MDR *E. coli* in finishing pigs than in weaned or suckling pigs [36,37]. A higher prevalence of MDR in weaned piglets is usually explained by the wider use of antibiotics for treatment and prophylaxis because younger animals have an increased risk of enteric infections, especially when stressed by weaning and mixing litters [36]. Sows received antibiotics more often by parenteral administration [38], which would be a positive factor for the spread of resistance, but, at the same time, different types of active ingredients of antimicrobials were chosen more often [39], which could contribute to the high prevalence of MRD bacteria.

Among ESBL-producing *E. coli* isolates, *bla*_TEM_ was found to be the most predominant, although it was only 8% more present than *bla*_CTX-M_. The distribution of ESBL genes in isolates from food-production animals in Europe has been reported by Silva et al. [6]. In pigs, the most frequently observed *bla*_CTX-M_ genes were *bla*_CTX-M-1_ and *bla*_CTX-M-15_ from *bla*_CTX-M-group1_. The genes *bla*_CTX-M-group4_ and *bla*_CTX-M-group2_ were relatively less common with 5% (*bla*_CTX-M-14_) and 3% (*bla*_CTX-M-2_), respectively [6]. As *bla*_CTX-M-group2_ and *bla*_CTX-M-group4_ were not identified in our study, the prevalence of the *bla*_CTX–M_ genes could be slightly higher. A high prevalence of *bla*_CTX-M-group1_ and *bla*_TEM_ in pigs was reported by other authors from diverse European countries, and similarly to our results, the genes *bla*_TEM_ and *bla*_CTX-M_ were most commonly observed in combination [40,41,42].

Our results demonstrated the differences in the distribution of beta-lactamase genes on the large and small farms. The gene of *bla*_CTX-M_ in combination with other beta-lactamase genes were observed considerably more frequently on the large farms, while the gene of *bla*_SHV_ in combination with other beta lactamase genes was dominant on the small farm. The main reason for the higher prevalence of *bla*_CTX-M_ on the large farms could be the zinc oxide added to the feed. A recent study showed that the addition of zinc to fecal suspension in vitro increased the proportion of a plasmid-encoded *bla*_CTX-M-1_ *E. coli* compared with the total microbiota. In addition, zinc may induce the expression of resistance genes in the *E. coli* strains [43]. On the large farms, zinc was probably responsible for the spread of plasmid-encoded ESBL strains and inducing the expression of the *bla*_CTX-M_ gene. This may explain the characteristic phenotype of *bla*_CTX-M_ against several beta lactam antibiotics—cefazolin, cephalexin, cefotaxime and cefepime.

We observed a positive association between the gene *bla*_SHV_ and the resistance of amoxicillin-clavulanic acid, ceftazidime and cefixime. However, the gene *bla*_CTX-M_ had a positive association with the resistance of cephalexin and several non-beta-lactam antibiotics—enrofloxacin, tetracycline, gentamicin, trimethoprim and sulfamethoxazole-trimethoprim. Some TEM and SHV mutants have a single amino acid change at the amber position, therefore they overtake the first generation (e.g., clavulanic acid) of beta-lactamase inhibitors [44,45]. Other authors have reported the positive association of SHV with resistance to ceftazidime due to the higher hydrolytic activity of SHV against ceftazidime than against cefotaxime, which is attributed to the Glu^240^ Lys substitution [27]. It has not been clearly defined whether the presence of *bla*_SHV_ specifically causes resistance to cefixime. Moreover, Naziri at al. [46] have reported a high resistance to cefixime equally often in both isolates that contain *bla*_CTX-M_ or *bla*_SHV_. It should be noted that Livermore at al. [47] reported that the level of resistance to the oral cephalosporin (cefixime) depends on the specific enzyme variant. A high degree of resistance was observed in SHV-4, SHV-5, CTX-M-15, but low resistance was observed in SHV-2 and CTX-M-9. This suggests the presence of specific SHV enzymes with an increased activity against cefixime; further in-depth studies would be required for confirmation. In our study, resistance to cephalexin showed a positive association with *bla*_CTX-M_, but a negative association with *bla*_SHV_. Broad spectrum beta-lactamases are usually especially active against the first-generation cephalosporins [48], which explained the positive association with the resistance of cephalexin. Interestingly, our results showed that cephalexin was effective against SHV. Similarly, Bedenic at al. [49] have reported good activity of cephalexin against SHV-2. Observation of the positive association of *bla*_CTX-M_ with several non-beta lactam antibiotics could indicate the prevalence of specific MDR ESBL clones. Although all of ESBL genes may be located on the mobile genetic elements and are often associated with co-resistance phenotypes with aminoglycosides, fluoroquinolones, trimethoprim-sulphamethoxazole, tetracycline and other antibiotics [50,51], *bla*_CTX-M_ is most commonly associated with broad MDR strains [29]. Clonal dispersion and the co-selection processes of CTX-M clones promote their spread globally as they are easily transmitted among family members in a colonization [52]. In recent years, there has been concern about the coexistence of *bla*_CTX-M_ and plasmid-mediated-quinolone-resistance (PMQR), therefore non-discriminatory use of fluoroquinolone may result in the selection and rapid dissemination of MDR ESBLs in commensal *E. coli* [24]. Furthermore, a strong association of PMQR and *bla*_CTX-Mgroup1_ (especially *bla*_CTX-M-15_) was observed among ESBL-producing *E. coli* [53,54]. Dietary zinc for weaned piglets also promotes the abundance of MDR and might promote the selective growth and expression of plasmid-encoded ESBL-producing *E. coli* [43]. The above factors were responsible for the spread of MDR CTX-M strains on the large farms.

## 4. Materials and Methods

### 4.1. Sample Collection and Characterization of Farms

Fecal samples (n = 615) were collected from 4-week, 5-week, 6-week, 8-week, 12-week and 20-week-old piglets, pigs and sows from four large (L1, L2, L3, L4) and three small (S1, S2, S3) farrow-to-finish pig farms in Latvia. On farms L1, L2, L3, L4, S1, S2 and S3, the total number of the sows was 2100, 700, 1700, 1000, 15, 40, 20, respectively. A more detailed characterization of the farms is given in Table 4. Two of four randomly selected fecal samples were collected in sterile Whirl-Pack bags in each pig pen, depending on the circumstances: immediately after defecation or choosing as recently obtained fecal masses as possible. The samples were transported and stored at 2–8 °C, and the bacteriological examination was started within 24 h.

### 4.2. Screening of Extended-Spectrum-Beta-Lactamase-Producing E. coli

The screening of ESBL-producing *E. coli* was performed according to the following method: a directly inoculated sample was placed on the selected media [55,56] Briefly, using a sterile cotton swab, the fecal specimen was gently spread onto the ChromArt ESBL agar (Biolife, Milano, Italy) surface. The plates were incubated at 36 °C for 20–24 h. If the plates were negative, they were re-incubated for an additional 24 h. After the incubation, the large pink colonies were noted and regarded as presumptive ESBL producing *E. coli.* One typical presumptive ESBL colony was sub-cultured on a Tryptic soy agar (Biolife, IT) and incubated overnight at 36 °C to obtain pure culture for the identification and phenotypic confirmatory test. The oxidase negative, indole positive, urease and citrate negative colonies were considered to be *E. coli*, and the isolates were confirmed by VITEK MS (bioMerieux SA, Marcy L’Etoile, France) which uses MALDI-TOF technology.

### 4.3. Phenotypic Confirmatory Test for ESBLs in E. coli

According to EUCAST recommendations [57], the combination disc test (CDT) was chosen for the confirmation of ESBL. The CDT was performed in accordance with the Clinical and Laboratory Standards Institute guidelines [58]. A standardized inoculum (0,5McF) of the presumptive ESBL-producing *E. coli* isolate was swabbed on the surface of Muller Hinton agar II (Biolife, IT). Cefotaxime 30 μg (CTX-30, BD BBL) and ceftazidime 30 μg (CAZ-30, BD BBL) discs alone and in combination with clavulanic acid 10 μg (CTX CLA and CAZ CLA, Biolife) were placed on the plate. The results were obtained after 18 h incubation at 36 °C. ESBL-producing *E. coli* was confirmed as positive if there was a ≥5 mm increase in the inhibition zone. This was detected by clavulanic acid around either the cefotaxime or the ceftazidime disc, compared to the diameter around the disc containing cefotaxime or ceftazidime alone. *E. coli* ATCC 25922 (Bioscience, Botolph Claydon, United Kingdom) was used for the quality control when the ESBL confirmatory test was carried out.

### 4.4. Antimicrobial Susceptibility Testing

For an in-depth analysis of resistance and molecular studies, 50 fecal ESBL-producing *E. coli* isolates from 4-week, 5-week, 6-week, 8-week, 12-week, and 20-week-old piglets, pigs and sows were selected by the stratified random sampling method and divided into three large groups: “sows”; 4-week, 5-week and 6-week-old piglets, referred to as “weaning-nursery”; and 8-week, 12-week and 20-week-old pigs, referred to as “growing-finishing”. The antimicrobial susceptibility of ESBL-producing *E. coli* isolates was determined according to the disc diffusion method [59] using antimicrobial susceptibility test discs (BD BBL Sensi-Disc, ASV) in a total of 18 samples. Eleven of these had beta-lactam group antibiotics: ampicillin (AM, 10 μg), mecillinam (MEL, 10 μg), ticarcillin (TIC, 75 μg), amoxicillin-clavulanic acid (AMC, 20 μg /10 μg), cephalexin (CL, 30 μg), cefazolin (CZ, 30 μg), cefoxitin (FOX, 30 μg), ceftazidime (CAZ, 30 μg), cefotaxime (CTX, 30 μg), cefixime (CFM 5 μg), cefepime (FEP, 30 μg), imipenem (IMP, 10 μg), while the other seven had non-beta-lactam group antibiotics: sulfamethoxazole-trimethoprim (SXT, 23.75 μg /1.25 μg), trimethoprim (TMP, 5 μg), gentamicin (GM, 10 μg), chloramphenicol (C, 30 μg), tetracycline (TE, 30 μg), enrofloxacin (ENO, 5 μg), ciprofloxacin (CIP, 5 μg). After incubation, the zone (mm) of inhibition was interpreted using the breakpoint tables according to EUCAST (2019) guidelines [60]. Enrofloxacin was not included in the breakpoint table, and therefore it was interpreted according to the manufacturer’s recommendations. Based on the directions of Magiorakos et al. [14], ESBL-producing *E. coli* was defined as MDR if the isolate had non-susceptibility to at least one antimicrobial agent in ≥3 antimicrobial categories [14]. Different levels of MDR were defined according to the ones described before [61]

### 4.5. Identification of Beta-Lactamase Genes in the Fecal ESBL-Producing E. coli

DNA was extracted using the E.Z.N.A. Bacterial DNA kit (Omega Bio-tek, Norcross, GA, USA) according to the procedure given by the manufacturer [62]. The concentration of DNA was estimated by the ND-1000 Spectrophotometer. The polymerase chain reaction was carried out by HotStarTaq^®^ Plus Master Mix Kit (QIAGEN, Hilden, Germany) according to the procedure given by the manufacturer.

The identification of the *bla*_TEM_ gene was conducted with primers TEM forward (5′-AGTGCTGCCATAACCATGAGTG-3′) and TEM reverse (5′-CTGACTCCCCGTCGTGTAGATA-3′). The primers for amplification of the *bla*_SHV_ gene were SHV forward (5′–GATGAACGCTTTCCCATGATG-3′) and SHV reverse (5′-CGCTGTTATCGCTCATGGTAA-3′), but for the identification of *bla*_CTX-M,_ gene primers CTX-M-group1 forward (5′–TCCAGAATAAGGAATCCCATGG-3′ and CTX-M-group1 reverse (5′-TGCTTTACCCAGCGTCAGAT-3′) were used.

The amplification reactions were performed in the Applied Biosystems 2720 thermal cycler under the following conditions: initial denaturation at 95 °C for 5 min, denaturation at 94 °C for 1 min of 35 cycles, annealing at 55 °C for 1 min for *bla*_TEM_, at 53 °C for 1 min for *bla*_CTX-M_, at 47 °C for 1 min for *bla*_SHV_, then extension at 72 °C for 1 min, followed by the final extension at 72 °C for 10 min.

After the amplification, PCR products were separated by a 2% agarose gel. After electrophoresis, the amplified DNA fragments were visualized by UV transilluminator and the gels were photographed. The amplicon size for *bla*_TEM_ was 431 bp, for *bla*_SHV_ it was 214 bp and for *bla*_CTX-M_ it was 621 bp.

### 4.6. Statistical Analysis

The statistical analysis was conducted by R Studio software (version 1.1.463). The Chi square test was used to compare the significance of differences in the prevalence of ESBL-producing *E. coli* between the large and small farms. The pairwise comparisons from the Chi-squared test were used for post hoc identification of significant differences in the prevalence of ESBL-producing *E. coli* in different farms and in different age groups of pigs. Fifty of ESBL-producing *E. coli* isolates were selected by the stratified random sampling method for in-depth studies of antimicrobial resistance and beta-lactamase genes. The Fisher’s exact test was used to compare the distribution and proportion of antimicrobial resistance and beta-lactamase genes. Odds ratios and 95% confidence intervals were used to evaluate the associations between beta-lactamase genes and antibiotic resistance. Statistical significance was considered as *p* < 0.05

## 5. Conclusions

The prevalence of ESBL-producing *E. coli* varies widely among farms and although higher prevalence was observed in the large farms, small farms are also of concern. The screening of ESBL-producing *E. coli* demonstrated a high prevalence of *bla*_CTX-M_ with extended MDR phenotypes on large farms, but the dominance of *bla*_SHV_ may also cause trouble due to the high resistance to ceftazidime and cefixime on small farms. Various contributing factors such as piglet diarrhea, the broad use of amoxicillin, fluoroquinolones and zinc included as a feed additive may contribute to a number of hazardous ESBL clones on farms. Individual analysis of the antibiotic resistance situation on small farms could be an important step for revealing hazardous MDR ESBL strains and to review the antimicrobial therapy in use. Large farms are more exposed to the alarming spread of MDR ESBL, which emphasizes the need to limit the use of antimicrobial therapy and the urgency of finding alternative strategies.

## Figures and Tables

**Table 1:** Prevalence of ESBL-producing *E. coli* on pig farms.

Site		Positive
**Presence of *ESBL*-producing *E. coli* on large and small pig farms, (%)**		
Large farms (n = 465)	L1 (n = 115)	74 (64.3) ^c^
	L2 (n = 127)	38 (29.9) ^b^
	L3 (n = 121)	13 (10.7) ^a^
	L4 (n = 102)	0
Small farms (n = 150)	S1 (n = 40)	19 (47.5) ^c^
	S3 (n = 41)	0
	S2 (n = 69)	0
**Presence of ESBL-producing *E. coli* in pigs of different age, (%)**		
Weeks of age (n = 538)	4 (n = 110)	31 (28.2) ^b^
	6 (n = 151)	53 (35.1) ^b^
	8 (n = 105)	11 (10.5) ^a^
	12 (n = 87)	10 (11.5) ^a^
	20 (n = 85)	7 (8.2) ^a^
Sows (n = 77)		32 (41.6) ^b^

^a,b,c^—mean values in the same column with different letters are significantly different (*p* < 0.05).

**Table 2:** Occurrence of pattern for resistance to beta lactams antibiotics and beta-lactamase gene(s) detected in ESBL-producing *E. coli*. Note: AM–ampicillin, TIC–ticarcillin, AMC–amoxicillin-clavulanic acid, CL–cefalexin, CZ–cefazolin, FOX–cefoxitin, CAZ–ceftazidime, CTX–cefotaxime, CFM–cefixime, FEP–cefepime.

Patterns of Resistance to Beta-lactams	Number of Beta-lactamase Gene(s)						
	*bla*_CTX-M_	*bla*_SHV_	*bla*_TEM_	*bla*_TEM+_ *bla*_SHV_	*bla*_TEM+_ *bla*_CTX-M_	*bla*_TEM+_ *bla*_SHV+_ *bla*_CTX-M_	Total
**Large farms (L1, L2, L3)**							
AM-TIC-AMC-CL-CZ-CAZ-CTX-CFM-FEP	0	0	0	0	1	1	2
AM-TIC-AMC-CL-CZ-CTX-CFM-FEP	1	0	1	0	6	9	17
AM-TIC-AMC-CL-CZ-CTX-FEP	1	0	0	0	8	4	13
AM-TIC-AMC-CL-CZ-FOX-CTX-CFM-FEP	1	0	0	0	0	0	1
AM-TIC-CL-CZ-CTX-CFM-FEP	0	0	0	0	1	0	1
AM-TIC-CL-CZ-CTX-FEP	0	0	0	0	4	0	4
**Small farm (S1)**							
AM-TIC-AMC-CZ-CAZ-CTX-CFM	0	0	0	1	0	3	4
AM-TIC-AMC-CZ-CAZ-CTX-CFM-FEP	0	0	0	4	1	1	6
AM-TIC-CZ	0	0	1	0	0	1	2
Total	3	0	2	5	21	19	50

**Table 3:** Statistically significant associations between β-lactamase gene and antimicrobial resistance in ESBL-producing *E. coli* isolates.

Selected Beta-Lactamase Gene	Antibiotic	OR	95% CI	*p*-Value
*bla*_SHV_	AMC	6.79	1.39–66.10	0.0083
	CL	0.12	0.03–0.41	0.0001
	CAZ	8.38	2.47–37.17	0.0001
	CFM	4.36	1.70–12.00	0.0009
	FEP	0.15	0.01–0.79	0.0124
	SXT	0.30	0.12–0.77	0.0067
	TMP	0.30	0.12–0.77	0.0067
*bla*_CTX-M_	CL	34.86	6.63–357.80	0.0001
	CTX	6.77	0.45–101.68	0.0931
	CAZ	0.08	0.01–0.33	0.0001
	SXT	15.03	3.02–148.12	0.0001
	TMP	15.03	3.02–148.12	0.0001
	GM	inf	2.90–inf	0.0003
	TE	12.11	2.45–118.82	0.0003
	ENO	inf	1.46–inf	0.0093

**Table 4:** Characteristics of farms, prophylaxis and antimicrobial policy. AMX–amoxicillin, AMC–amoxicillin + clavulanic acid, CFT–ceftiofur, CFQ–cefquinome, CS–colistin, ENO–enrofloxacin, GM–gentamicin, OT–oxytetracycline, P–penicillin, MAR–marbofloxacin, TM–tiamulin, TY–tylosin, TMP + S–trimethoprim + sulfadiazine, ZnO–Zinc oxide.

Farm	Total Number of Sows	Time to Weaning (Days)	Diarrhea/Critical Periods	Feed Additives	Antibiotics Used			Immunisation against *E. coli ^b^*
					Penicillin/Board-Spectrum Penicillins	Cephalo-Sporins	Non-Beta Lactams	
L1	2100	28	common/ 1–3 days; 4–6 weeks; 7–8 weeks	ZnO–150 mg kg^−1^	AMX	CFT	CS, ENO, MAR, OT, SXT ^a^	yes
L2	700	33	common/ 2–3 weeks; 5–6 weeks	ZnO–150 mg kg^−1^; during weaning–zinc and electrolyte agent ^c^	P, AMX	no	GM, OT, TMP + S,	yes
L3	1700	28	sporadically	ZnO–150 mg kg^−1^; starter phase (2–3 weeks after weaning)–ZnO 2500 mg kg^−1^; water acidification ^d^	no	CFT, CFQ	CS, ENO, GM, OT, TMP + S,	yes
L4	1000	28	sporadically	ZnO–150 mg kg^−1^;	P, AMC	no	OT TMP + S,	no
S1	15	28	common/ 5–6 weeks	-	P, AMX	no	ENO, OT, TMP + S	yes
S2	40	28–42	rare	-	no	no	TY	no
S3	20	40	rare	-	no	no	TM	no

^a^ The antibiotic is changed every three months to ensure the effectiveness of the antimicrobial drugs. ^b^ For prevention of different strains of *E. coli* infections, for the passive immunization of progeny (piglets) by active immunization of sows and gilts, “Porcilis ColiClos” is used (AN Boxmeer, Netherlands), ^c^ “Revifit (veromin)” (Agro-strefa, Poland), ^d^ “Baracid”, (Fermo, Poland).

## Data Availability

The data used to support the findings of this study are available from the corresponding author upon request.

## References

[1] EDCD, EFSA, EMA (2017). ECDC/EFSA/EMA Second Joint Report on the Integrated Analysis of the Consumption of Antimicrobial Agents and Occurrence of Antimicrobial Resistance in Bacteria from Humans and Food-producing Animals. EFSA J..

[2] Van Boeckel T.P., Brower C., Gilbert M., Grenfell B.T., Levin S.A., Robinson T.P., Teillant A., Laxminarayan R. (2015). Global Trends in Antimicrobial Use in Food Animals. Proc. Natl. Acad. Sci. USA.

[3] Maes D.G.D., Dewulf J., Piñeiro C., Edwards S., Kyriazakis I. (2020). A Critical Reflection on Intensive Pork Production with an Emphasis on Animal Health and Welfare. J. Anim. Sci..

[4] EFSA, ECDC (2020). The European Union Summary Report on Antimicrobial Resistance in Zoonotic and Indicator Bacteria from Humans, Animals and Food in 2017/2018. EFSA J..

[5] ESVAC (2020). Sales of Veterinary Antimicrobial Agents in 31 European Countries in 2018.

[6] Silva N., Carvalho I., Currie C., Sousa M., Igrejas G., Poeta P., Capelo-Martinez J.-L., Igrejas G. (2019). Extended-Spectrum-β-Lactamase and Carbapenemase-Producing Enterobacteriaceae in Food-Producing Animals in Europe: An impact on public health?. Antibiotic Drug Resistance.

[7] Carvalho I., Cunha R., Martins C., Martínez-Álvarez S., Safia Chenouf N., Pimenta P., Pereira A.R., Ramos S., Sadi M., Martins Â. (2021). Antimicrobial Resistance Genes and Diversity of Clones among Faecal ESBL-Producing *Escherichia coli* Isolated from Healthy and Sick Dogs Living in Portugal. Antibiotics.

[8] Ramos S., Silva V., Dapkevicius M.d.L.E., Caniça M., Tejedor-Junco M.T., Igrejas G., Poeta P. (2020). *Escherichia coli* as Commensal and Pathogenic Bacteria among Food-Producing Animals: Health Implications of Extended Spectrum β-Lactamase (ESBL) Production. Animals.

[9] WHO (2017). Prioritization of Pathogens to Guide Discovery, Research and Development of New Antibiotics for Drug Resistant Bacterial Infections, Including Tuberculosis.

[10] Bergšpica I., Kaprou G., Alexa E.A., Prieto M., Alvarez-Ordóñez A. (2020). Extended Spectrum β-Lactamase (ESBL) Producing *Escherichia coli* in Pigs and Pork Meat in the European Union. Antibiotics.

[11] De Koster S., Ringenier M., Lammens C., Stegeman A., Tobias T., Velkers F., Vernooij H., Kluytmans-van den Bergh M., Kluytmans J., Dewulf J. (2021). ESBL-Producing, Carbapenem- and Ciprofloxacin-Resistant *Escherichia coli* in Belgian and Dutch Broiler and Pig Farms: A Cross-Sectional and Cross-Border Study. Antibiotics.

[12] van der Fels-Klerx H.J., Puister-Jansen L.F., van Asselt E.D., Burgers S.L.G.E. (2011). Farm Factors Associated with the Use of Antibiotics in Pig Production1. J. Anim. Sci..

[13] Ström G., Halje M., Karlsson D., Jiwakanon J., Pringle M., Fernström L.-L., Magnusson U. (2017). Antimicrobial Use and Antimicrobial Susceptibility in *Escherichia coli* on Small- and Medium-Scale Pig Farms in North-Eastern Thailand. Antimicrob. Resist. Infect. Control.

[14] Magiorakos A.-P., Srinivasan A., Carey R.B., Carmeli Y., Falagas M.E., Giske C.G., Harbarth S., Hindler J.F., Kahlmeter G., Olsson-Liljequist B. (2012). Multidrug-Resistant, Extensively Drug-Resistant and Pandrug-Resistant Bacteria: An International Expert Proposal for Interim Standard Definitions for Acquired Resistance. Clin. Microbiol. Infect..

[15] Dohmen W., Bonten M.J.M., Bos M.E.H., van Marm S., Scharringa J., Wagenaar J.A., Heederik D.J.J. (2015). Carriage of Extended-Spectrum β-Lactamases in Pig Farmers Is Associated with Occurrence in Pigs. Clin. Microbiol. Infect..

[16] Moor J., Aebi S., Rickli S., Mostacci N., Overesch G., Oppliger A., Hilty M. (2021). Dynamics of Extended-Spectrum Cephalosporin-Resistant *Escherichia coli* in Pig Farms: A Longitudinal Study. Int. J. Antimicrob. Agents.

[17] Schmid A., Hörmansdorfer S., Messelhäusser U., Käsbohrer A., Sauter-Louis C., Mansfeld R. (2013). Prevalence of Extended-Spectrum β-Lactamase-Producing *Escherichia coli* on Bavarian Dairy and Beef Cattle Farms. Appl. Environ. Microbiol..

[18] Schmithausen R.M., Schulze-Geisthoevel S.V., Stemmer F., El-Jade M., Reif M., Hack S., Meilaender A., Montabauer G., Fimmers R., Parcina M. (2015). Analysis of Transmission of MRSA and ESBL-E among Pigs and Farm Personnel. PLoS ONE.

[19] Dohmen W., Dorado-García A., Bonten M.J.M., Wagenaar J.A., Mevius D., Heederik D.J.J. (2017). Risk Factors for ESBL-Producing *Escherichia coli* on Pig Farms: A Longitudinal Study in the Context of Reduced Use of Antimicrobials. PLoS ONE.

[20] Gay N., Leclaire A., Laval M., Miltgen G., Jégo M., Stéphane R., Jaubert J., Belmonte O., Cardinale E. (2018). Risk Factors of Extended-Spectrum β-Lactamase Producing Enterobacteriaceae Occurrence in Farms in Reunion, Madagascar and Mayotte Islands, 2016–2017. Vet. Sci..

[21] Reyns T., De Boever S., Schauvliege S., Gasthuys F., Meissonnier G., Oswald I., De Backer P., Croubels S. (2009). Influence of Administration Route on the Biotransformation of Amoxicillin in the Pig. J. Vet. Pharmacol. Ther..

[22] Burch D.G.S., Sperling D. (2018). Amoxicillin-Current Use in Swine Medicine. J. Vet. Pharmacol. Ther..

[23] Fournier C., Aires-de-Sousa M., Nordmann P., Poirel L. (2020). Occurrence of CTX-M-15- and MCR-1-Producing Enterobacterales in Pigs in Portugal: Evidence of Direct Links with Antibiotic Selective Pressure. Int. J. Antimicrob. Agents.

[24] Basu S., Mukherjee M. (2018). Incidence and Risk of Co-Transmission of Plasmid-Mediated Quinolone Resistance and Extended-Spectrum β-Lactamase Genes in Fluoroquinolone-Resistant Uropathogenic *Escherichia coli*: A First Study from Kolkata, India. J. Glob. Antimicrob. Resist..

[25] Livermore D.M., Day M., Cleary P., Hopkins K.L., Toleman M.A., Wareham D.W., Wiuff C., Doumith M., Woodford N. (2019). OXA-1 β-Lactamase and Non-Susceptibility to Penicillin/β-Lactamase Inhibitor Combinations among ESBL-Producing *Escherichia coli*. J. Antimicrob. Chemother..

[26] Rawat D., Nair D. (2010). Extended-Spectrum β-Lactamases in Gram Negative Bacteria. J. Glob. Infect. Dis..

[27] Liakopoulos A., Mevius D., Ceccarelli D. (2016). A Review of SHV Extended-Spectrum β-Lactamases: Neglected Yet Ubiquitous. Front. Microbiol..

[28] Bonnet R. (2004). Growing Group of Extended-Spectrum β-Lactamases: The CTX-M Enzymes. Antimicrob. Agents Chemother..

[29] Zeynudin A., Pritsch M., Schubert S., Messerer M., Liegl G., Hoelscher M., Belachew T., Wieser A. (2018). Prevalence and Antibiotic Susceptibility Pattern of CTX-M Type Extended-Spectrum β-Lactamases among Clinical Isolates of Gram-Negative Bacilli in Jimma, Ethiopia. BMC Infect. Dis..

[30] Garcia-Graells C., Berbers B., Verhaegen B., Vanneste K., Marchal K., Roosens N.H.C., Botteldoorn N., De Keersmaecker S.C.J. (2020). First Detection of a Plasmid Located Carbapenem Resistant BlaVIM-1 Gene in *E. coli* Isolated from Meat Products at Retail in Belgium in 2015. Int. J. Food Microbiol..

[31] Prescott J.F., Giguère S., Prescott J.F., Dowling P.M. (2013). Beta-lactam Antibiotics. Antimicrobial Therapy in Veterinary Medicine.

[32] Fuchs F., Hamprecht A. (2019). Results from a Prospective In Vitro Study on the Mecillinam (Amdinocillin) Susceptibility of Enterobacterales. Antimicrob. Agents Chemother..

[33] EMA, CVMP, CHMP (2019). Categorisation of Antibiotics in the European Union.

[34] Hölzel C.S., Müller C., Harms K.S., Mikolajewski S., Schäfer S., Schwaiger K., Bauer J. (2012). Heavy Metals in Liquid Pig Manure in Light of Bacterial Antimicrobial Resistance. Environ. Res..

[35] Ciesinski L., Guenther S., Pieper R., Kalisch M., Bednorz C., Wieler L.H. (2018). High Dietary Zinc Feeding Promotes Persistence of Multi-Resistant *E. coli* in the Swine Gut. PLoS ONE.

[36] Akwar H.T., Poppe C., Wilson J., Reid-Smith R.J., Dyck M., Waddington J., Shang D., McEwen S.A. (2008). Prevalence and Patterns of Antimicrobial Resistance of Fecal *Escherichia coli* among Pigs on 47 Farrow-to-Finish Farms with Different in-Feed Medication Policies in Ontario and British Columbia. Can. J. Vet. Res. = Rev. Can. Rech. Vet..

[37] De Lucia A., Card R.M., Duggett N., Smith R.P., Davies R., Cawthraw S.A., Anjum M.F., Rambaldi M., Ostanello F., Martelli F. (2021). Reduction in Antimicrobial Resistance Prevalence in *Escherichia coli* from a Pig Farm Following Withdrawal of Group Antimicrobial Treatment. Vet. Microbiol..

[38] Jensen V.F., Emborg H.-D., Aarestrup F.M. (2012). Indications and Patterns of Therapeutic Use of Antimicrobial Agents in the Danish Pig Production from 2002 to 2008. J. Vet. Pharmacol. Ther..

[39] Lekagul A., Tangcharoensathien V., Mills A., Rushton J., Yeung S. (2020). How Antibiotics Are Used in Pig Farming: A Mixed-Methods Study of Pig Farmers, Feed Mills and Veterinarians in Thailand. BMJ Glob. Health.

[40] Barilli E., Vismarra A., Villa Z., Bonilauri P., Bacci C. (2019). ESβL *E. coli* Isolated in Pig’s Chain: Genetic Analysis Associated to the Phenotype and Biofilm Synthesis Evaluation. Int. J. Food Microbiol..

[41] Biasino W., De Zutter L., Garcia-Graells C., Uyttendaele M., Botteldoorn N., Gowda T., Van Damme I. (2018). Quantification, Distribution and Diversity of ESBL/AmpC-Producing *Escherichia coli* on Freshly Slaughtered Pig Carcasses. Int. J. Food Microbiol..

[42] Von Salviati C., Friese A., Roschanski N., Laube H., Guerra B., Käsbohrer A., Kreienbrock L., Roesler U. (2014). Extended-Spectrum Beta-Lactamases (ESBL)/AmpC Beta-Lactamases-Producing *Escherichia coli* in German Fattening Pig Farms: A Longitudinal Study. Berl. Munch. Tierarztl. Wochenschr..

[43] Peng S., Herrero-Fresno A., Olsen J.E., Dalsgaard A. (2020). Influence of Zinc on CTX-M-1 β-Lactamase Expression in *Escherichia coli*. J. Glob. Antimicrob. Resist..

[44] Cantón R., Morosini M.I., Martin O., de la Maza S., de la Pedrosa G.E.G. (2008). IRT and CMT β-Lactamases and Inhibitor Resistance. Clin. Microbiol. Infect..

[45] Bush K., Bradford P.A. (2019). Interplay between β-Lactamases and New β-Lactamase Inhibitors. Nat. Rev. Microbiol..

[46] Naziri Z., Derakhshandeh A., Soltani Borchaloee A., Poormaleknia M., Azimzadeh N. (2020). Treatment Failure in Urinary Tract Infections: A Warning Witness for Virulent Multi-Drug Resistant ESBL- Producing *Escherichia coli*. Infect. Drug Resist..

[47] Livermore D.M., Mushtaq S., Nguyen T., Warner M. (2011). Strategies to Overcome Extended-Spectrum β-Lactamases (ESBLs) and AmpC β-Lactamases in Shigellae. Int. J. Antimicrob. Agents.

[48] Paterson D.L., Bonomo R.A. (2005). Extended-Spectrum β-Lactamases: A Clinical Update. Clin. Microbiol. Rev..

[49] Bedenic B., Vranes J., Suto S., Zagar Z. (2005). Bactericidal Activity of Oral β-Lactam Antibiotics in Plasma and Urine versus Isogenic *Escherichia coli* Strains Producing Broad- and Extended-Spectrum β-Lactamases. Int. J. Antimicrob. Agents.

[50] Ozgumus O.B., Tosun I., Aydin F., Kilic A.O. (2008). Horizontol Dissemination of TEM- and SHV-Typr Beta-Lactamase Genes-Carrying Resistance Plasmids amongst Clonical Isolates of Enterobacteriaceae. Braz. J. Microbiol..

[51] Balkhed Å.Ö., Tärnberg M., Monstein H.-J., Hällgren A., Hanberger H., Nilsson L.E. (2013). High Frequency of Co-Resistance in CTX-M-Producing *Escherichia coli* to Non-Beta-Lactam Antibiotics, with the Exceptions of Amikacin, Nitrofurantoin, Colistin, Tigecycline, and Fosfomycin, in a County of Sweden. Scand. J. Infect. Dis..

[52] Cantón R., González-Alba J.M., Galán J.C. (2012). CTX-M Enzymes: Origin and Diffusion. Front. Microbiol..

[53] Salah F.D., Soubeiga S.T., Ouattara A.K., Sadji A.Y., Metuor-Dabire A., Obiri-Yeboah D., Banla-Kere A., Karou S., Simpore J. (2019). Distribution of Quinolone Resistance Gene (Qnr) in ESBL-Producing *Escherichia coli* and *Klebsiella* spp. in Lomé, Togo. Antimicrob. Resist. Infect. Control.

[54] Kim J.O., Yoo I.Y., Yu J.K., Kwon J.A., Kim S.Y., Park Y.-J. (2021). Predominance and Clonal Spread of CTX-M-15 in Cefotaxime-Resistant Klebsiella Pneumoniae in Korea and Their Association with Plasmid-Mediated Quinolone Resistance Determinants. J. Infect. Chemother..

[55] Lalak A., Wasyl D., Zając M., Skarżyńska M., Hoszowski A., Samcik I., Woźniakowski G., Szulowski K. (2016). Mechanisms of Cephalosporin Resistance in Indicator *Escherichia coli* Isolated from Food Animals. Vet. Microbiol..

[56] Blanc D.S., Poncet F., Grandbastien B., Greub G., Senn L., Nordmann P. (2021). Evaluation of the Performance of Rapid Tests for Screening Carriers of Acquired ESBL-Producing Enterobacterales and Their Impact on Turnaround Time. J. Hosp. Infect..

[57] EUCAST (2017). EUCAST Guidelines for Detection of Resistance Mechanisms and Specific Resistances of Clinical and/or Epidemiological Importance; Version 2.0. https://www.eucast.org/resistance_mechanisms/.

[58] CLSI (2018). Performance Standards for Antimicrobial & Antifungal Susceptibility Testing. Clinical and Laboratory Standards Institute, Wayne, USA, M100 ED28:2018. https://clsi.org/about/about-clsi/about-clsi-antimicrobial-and-antifungal-susceptibility-testing-resources/.

[59] EUCAST (2017). EUCAST Disk Diffusion Method for Antimicrobial Susceptibility Testing, Version 6.0. https://kaldur.landspitali.is/focal/gaedahandbaekur/gnhsykla.nsf/5e27f2e5a88c898e00256500003c98c2/795d21eac4c71fdf00257ac30056d723/$FILE/Manual_v_6.0_EUCAST_Disk_Test_final.pdf.

[60] EUCAST (2019). The European Committee on Antimicrobial Susceptibility Testing. Breakpoint Tables for Interpretation of MICs and Zone Diameters. Version 9.0. http://www.Eucast.org.

[61] Jahanbakhsh S., Smith M.G., Kohan-Ghadr H.-R., Letellier A., Abraham S., Trott D.J., Fairbrother J.M. (2016). Dynamics of Extended-Spectrum Cephalosporin Resistance in Pathogenic *Escherichia coli* Isolated from Diseased Pigs in Quebec, Canada. Int. J. Antimicrob. Agents.

[62] Kaftandzieva A., Trajkovska-Dokic E., Panovski N. (2011). Prevalence and Molecular Characterization of Extended Spectrum Beta-Lactamases (ESBLs) Producing *Escherichia coli* and Klebsiella Pneumoniae. Prilozi.

